# Abnormal Dynamic Functional Connectivity in Patients With End-Stage Renal Disease

**DOI:** 10.3389/fnins.2022.852822

**Published:** 2022-05-20

**Authors:** Xuekun Li, Ruifang Yan, Zheng Yue, Meng Zhang, Jipeng Ren, Baolin Wu

**Affiliations:** Department of Magnetic Resonance, First Affiliated Hospital of Xinxiang Medical University, Weihui, China

**Keywords:** end-stage renal disease, hemodialysis, resting-state functional magnetic resonance imaging, dynamic functional connectivity, graph theory

## Abstract

Dynamic functional connectivity (FC) analysis can capture time-varying properties of connectivity; however, studies focusing on dynamic FC in patients with end-stage renal disease (ESRD) are very limited. This is the first study to explore the dynamic aspects of whole-brain FC and topological properties in ESRD patients. Resting-state functional magnetic resonance imaging data were acquired from 100 ESRD patients [50 hemodialysis (HD) patients and 50 non-dialysis patients] and 64 healthy controls (HCs). Independent component analysis, a sliding-window approach and graph-theory methods were used to study the dynamic FC properties. The intrinsic brain FC were clustered into four configuration states. Compared with HCs, both patient groups spent longer time in State 3, in which decreased FC between subnetworks of the default mode network (DMN) and between the dorsal DMN and language network was observed in these patients, and a further reduction in FC between the DMN subnetworks was found in HD patients compared to non-dialysis patients. The number of transitions and the variability of global and local efficiency progressively decreased from that in HCs to that of non-dialysis patients to that of HD patients. The completion time of Trail Making Test A and Trail Making Test B positively correlated with the mean dwell time of State 3 and negatively correlated with the number of transitions in ESRD patients. Our findings suggest impaired functional flexibility of network connections and state-specific FC disruptions in patients with ESRD, which may underlie their cognitive deficits. HD may have an adverse effect on time-varying FC.

## Introduction

End-stage renal disease (ESRD) is the terminal stage of chronic kidney disease (i.e., category G5 according to international guidelines) (Webster et al., 2017). It is generally considered that the diagnosis of ESRD can be made when the estimated glomerular filtration rate (eGFR) drops below 15 ml/min per 1.73 m^2^ (Webster et al., 2017). Notably, 30–40% of ESRD patients on hemodialysis (HD) treatment exhibit cognitive dysfunction, especially impairments in the domains of orientation, attention and executive function (Krishnan and Kiernan, 2009; O’Lone et al., 2016). Cognitive impairment (CI) in those patients may contribute to long-term adverse consequences, including dementia and death, and is also associated with increased cost of medical care (Kurella Tamura and Yaffe, 2011; Williams et al., 2013). At present, the pathophysiology of CI in patients with ESRD who underwent HD has not been fully understood.

Resting-state functional magnetic resonance imaging (rs-fMRI) has become a valuable and non-invasive tool to investigate the pathophysiological mechanisms of multiple neurological and psychiatric diseases, such as major depression disorder (Wu et al., 2020b), Parkinson’s disease (Vo et al., 2017), and Alzheimer’s disease (Sheline and Raichle, 2013). It is reported that inherent brain activity in the resting state is spatially organized into a serious of functionally coherent patterns, namely resting-state networks (RSNs) (Damoiseaux et al., 2006; Jafri et al., 2008). A large number of rs-fMRI studies have been conducted to explore the pathophysiological bases of CI in patients with ESRD undergoing HD from the perspective of functional network connectivity. For example, using independent component analysis (ICA) algorithm, previous studies reported decreased functional connectivity (FC) in the default mode network (DMN) in patients with ESRD who received HD, and reduced DMN FC was associated with cognitive deficits in these patients (Ni et al., 2014; Lu et al., 2019). Furthermore, disrupted topological organization of whole-brain functional networks (Mu et al., 2018; Park et al., 2020; Wu et al., 2020a; Jin et al., 2021) and abnormal module-level interaction between the affective and cognitive control networks (Mu et al., 2018) have been found in HD patients. Dysfunctions in brain networks might contribute to the pathophysiological mechanism of CI in patients with ESRD who underwent HD. Overall, evidences from these studies suggest alterations in intrinsic brain functional networks, which may result in CI in ESRD patients treated with HD. However, all of the previous studies mainly focused on studying the static brain functional networks with the assumption that FC is constant during the entire rs-fMRI scanning, and the dynamic configuration of brain network connectivity is not taken into account.

Dynamic connectome, a new concept that focuses on the dynamic characteristics and patterns of brain networks, has attracted more and more attention. Using functional neuroimaging techniques, previous studies have identified the dynamic aspects of FC in normal subjects, and the dynamic FC was found to be related to higher-order cognitive domains (Kucyi et al., 2017; Shine et al., 2019; Soreq et al., 2019). In addition, altered dynamic FC configuration was found in patients with neurological and psychiatric disorders, such as major depressive disorder (Zhi et al., 2018; Xue et al., 2020), Parkinson’s disease (Kim et al., 2017), obsessive-compulsive disorder (Liu et al., 2021), and epilepsy (Liu et al., 2017). Additionally, dynamic FC analysis in combination with graph theory-based approach can also evaluate the variances in the graph metrics of time-varying brain connectivity and provide important imaging biomarkers underlying the pathophysiology of diseases. For example, a recent study found increased variability of global efficiency of the brain functional networks in patients with Parkinson’s disease compared with controls (Kim et al., 2017). On the contrary, increased variability of the connectivity strength, clustering coefficient and global efficiency was observed in patients with schizophrenia (Yu et al., 2015). However, alterations in whole-brain FC and network properties in the context of dynamic FC remain largely unknown in patients with ESRD. Up to now, only one study has been conducted to analyze resting-state FC dynamics in patients with ESRD. Based on a triple-network model [involving the salience network (SN), DMN, and central executive network], our recent study found that the dynamic FC within the triple networks was altered in HD patients compared to healthy controls (HCs) (Cao et al., 2021). Although this study demonstrated abnormal dynamic FC properties in HD patients, it only focused on the triple networks rather than the whole-brain networks, and the dynamic graph metrics which quantifiably describe the dynamic whole brain performance were not examined. More importantly, we did not evaluate the effect of HD on time-varying FC due to the lack of a non-dialysis positive control group.

Thus, by combining rs-fMRI data and a sliding-window approach, the purpose of this study was to investigate the differences of dynamic FC patterns between ESRD patients with and without HD. FC state analysis and a graph theory-based analysis were used to evaluate dynamic metrics. Based on the findings mentioned above, we hypothesized that (1) altered dynamic FC properties should be demonstrated in patients with ESRD; (2) HD might have an adverse effect on dynamic FC; and (3) some altered dynamic FC properties might correlate with cognitive performance in patients with ESRD.

## Materials and Methods

### Participants

This study was approved by the Institutional Review Board of the First Affiliated Hospital of Xinxiang Medical University. Written informed consent was obtained from all subjects prior to participation.

From October 2018 to May 2021, 110 patients with ESRD were recruited, including 56 patients undergoing HD (HD group) and 54 patients without any types of dialysis treatment (non-dialysis group). For study inclusion, all patients with ESRD were required to have a history of chronic glomerulonephritis with a disease duration of greater than 6 months, and all HD patients were required to have received regular dialysis treatment for more than 6 months. Exclusion criteria included: (a) history of any neurological diseases or psychiatric disorders, (b) presence of organic brain lesions detected by conventional MRI sequences, (c) history of traumatic brain injury, (d) contraindications to MR scanning, and (e) head movement greater than 1.5 mm or 1.5° or the mean framewise displacement (FD) larger than 0.2 mm during MR scanning. Based on these exclusion criteria, six HD patients were excluded due to brain infarcts (*n* = 1) and head motion more than 1.5 mm or 1.5° or the mean FD more than 0.2 (*n* = 5), and four non-dialysis patients were excluded due to a history of head trauma (*n* = 1) and head motion more than 1.5 mm or 1.5° or the mean FD more than 0.2 (*n* = 3). Consequently, the datasets from the remaining 50 HD patients (27 males and 23 females; mean age 36.10 ± 9.65) and 50 non-dialysis patients (26 males and 24 females; mean age 35.60 ± 9.06) were included in the final data analysis.

In addition, 64 healthy volunteers (HC group; 34 males and 30 females; mean age 34.56 ± 9.50) were recruited from the local community *via* advertisements. All of the healthy volunteers were more than 18 years old, and were required to have no diseases of the kidney, liver, or other organs and no history of neurological or psychiatric disorders. All patients with ESRD and HCs were righted-handed, and had normal sight to complete the neuropsychological tests. Some of the study samples were selected from our recently published papers, which mainly focused on investigating alterations in whole-brain functional network topologies (Wu et al., 2020a) and in dynamic FC properties within the triple networks (Cao et al., 2021) in patients with ESRD.

### Clinical Evaluation and Laboratory Examinations

One author (X.K.L.) reviewed the electronic medical records of all ESRD patients, and extracted demographic information such as dialysis duration and body mass index. To collect relevant blood biochemical indicators, blood biochemistry tests were conducted in all patients with ESRD 1 day before MR scanning.

### Neuropsychological Tests

One hour before MR scanning, all subjects underwent several neuropsychological tests, including the Mini-Mental State Examination (MMSE) (Folstein et al., 1975), Montreal Cognitive Assessment (MoCA) (Nasreddine et al., 2005), Trail Making Test A (TMT-A) (Bowie and Harvey, 2006), Trail Making Test B (TMT-B) (Bowie and Harvey, 2006), and Symbol Digit Modalities Test (SDMT) (Smith, 1982).

### Magnetic Resonance Imaging Data Acquisition

MR images were acquired using a 3.0-Tesla MR system (Discovery MR750, General Electric Healthcare, Milwaukee, WI) with a 16-channel head coil. During scanning, subjects were instructed to maintain their head still, keep their eyes closed, stay awake state, and avoid think of anything. First, conventional MR imaging sequences were acquired to detect the presence of brain organic lesions or any other abnormalities. Then, functional images were obtained using an echo-planar imaging sequence: 32 axial slices, repetition time (TR) = 2,000 ms, echo time (TE) = 41 ms, field of view (FOV) = 220 × 220 mm^2^, acquisition matrix = 64 × 64, section thickness = 4 mm, slice gap = 4.5 mm, flip angle = 90°, number of volumes = 180. High-resolution structural images were acquired using a brain volume sequence: 188 sagittal slices with thickness of 1 mm, inversion time = 450 ms, TR = 8.2 ms, TE = 3.2 ms, FOV = 256 × 256 mm^2^, matrix size = 256 × 256, flip angle = 12°.

### Image Preprocessing

Data preprocessing was conducted using the Data Processing and Analysis for Brain Imaging Toolbox (DPABI, version 4.1)^1^ (Yan et al., 2016) based on the MATLAB (version R2013b, MathWorks Inc., Natick, MA, United States) platform. For each subject’s rs-fMRI dataset, the first 10 volumes were removed to reduce equilibration effects. Slice-timing and head motion correction were conducted on the remaining rs-fMRI images. This realignment calculation generated a record of head motion within the entire rs-fMRI scanning. No significant difference in the mean FD (Jenkinson) values was found among the three groups (0.06 ± 0.04 for HD patients, 0.05 ± 0.03 for non-dialysis patients, and 0.05 ± 0.03 for HCs; *F* = 0.078, *p* = 0.925). A Diffeomorphic Anatomical Registration Through Exponentiated Lie Algebra (DARTEL) (Ashburner, 2007) algorithm was applied to register the functional images into the standard Montreal Neurological Institute (MNI) template with a re-sampled voxel size of 3 mm × 3 mm × 3 mm. Subsequently, the normalized brain functional images were spatially smoothed with a 6 mm full-width at half-maximum Gaussian kernel.

After data preprocessing, dynamic FC was analyzed according to the following steps: (1) identification of intrinsic connectivity networks, (2) computation for dynamic FC using a sliding window approach, (3) dynamic FC state analysis, and (4) dynamic graph theory analysis. A detailed overview of the framework is summarized in Supplementary Figure 1.

### Group Independent Component Analysis and Identification of Resting-State Networks

To decompose the preprocessed rs-fMRI data into different independent components (ICs), a spatial group ICA was performed using the Group ICA of fMRI Toolbox (GIFT, version 4.0b)^2^ (Calhoun et al., 2001). Although some studies selected a high-order ICA approach to obtain cortical and subcortical functional parcellations (Kim et al., 2017; Fiorenzato et al., 2019; Wang et al., 2021), we applied a low-order model, given the fact that the ICs obtained from the lower-order model were more consistent with previous anatomical and functional segmentations (Calhoun et al., 2008; Smith et al., 2009; Shi et al., 2018). Group ICA included the following steps: first, a two-step principal component analysis (subject-specific and group level) was used to decompose the data into 23 ICs. This averaged IC number was automatically estimated using the minimum description length criteria (Li et al., 2007). Second, ICA decomposition was performed using the Infomax algorithm (Bell and Sejnowski, 1995), and this step was repeated 20 times in ICASSO (Himberg et al., 2004) to obtain a stable and reliable set of 23 components. The group ICs of the 20 runs were clustered to assess their reliability, and only ICs with a higher average intra-cluster similarity [e.g., quality index (*I*_*q*_) > 0.7] were selected (Ma et al., 2011). Subsequently, the spatial maps and the time courses of BOLD signal were generated for each IC. Finally, a GICA back reconstruction algorithm (Calhoun et al., 2001) was used to back-project the group ICs. This step reconstructed subject-specific spatial maps and time courses for each IC.

**FIGURE 1:**
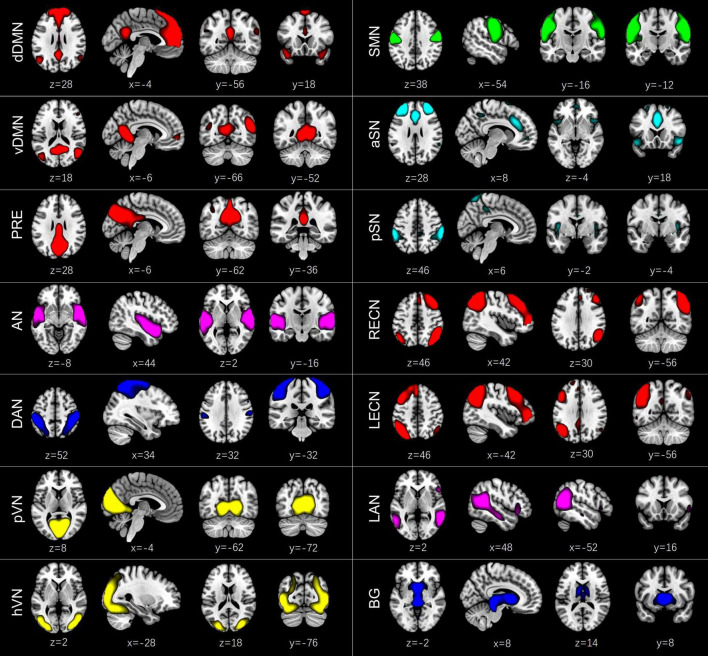
Spatial maps of the 14 independent components (ICs) identified by a group independent component analysis. These ICs were categorized into the dorsal default mode network (dDMN) (IC21), ventral default mode network (vDMN) (IC6), precuneus network (PRE) (IC5), auditory network (AN) (IC20), dorsal attention network (DAN) (IC8), primary visual network (pVN) (IC22), higher visual network (hVN) (IC16), sensorimotor network (SMN) (IC7), anterior salience network (aSN) (IC1), posterior salience network (pSN) (IC3), right executive control network (RECN) (IC18), left executive control network (LECN) (IC15), language network (LAN) (IC9), and basal ganglia network (BG) (IC4).

According to the following criteria (Cordes et al., 2000; Allen et al., 2014; Kim et al., 2017): (1) peak activations of spatial maps located in gray matter; (2) low spatial overlap with known vascular, ventricular, motion, and susceptibility artifacts; (3) time courses dominated by low frequency fluctuations and characterized by a high dynamic range, components were selected by calculating their spatial overlap with the Stanford functional ROIs template^3^ (Shirer et al., 2012), which contains 90 ROIs that belong to 14 predefined brain networks. A sorting function of spatial regression was used to estimate the similarity between the IC’s spatial image and a network template. An IC was assigned to a network if they showed the highest similarity, which was measured by the coefficient of determination (*R*^2^). Finally, we identified 14 ICs corresponding to different intrinsic connectivity networks: dorsal DMN (dDMN) (IC21), ventral DMN (vDMN) (IC6), precuneus network (PRE) (IC5), auditory network (AN) (IC20), dorsal attention network (DAN) (IC8), primary visual network (pVN) (IC22), higher visual network (hVN) (IC16), sensorimotor network (SMN) (IC7), anterior SN (aSN) (IC1), posterior SN (pSN) (IC3), right executive control network (RECN) (IC18), left executive control network (LECN) (IC15), language network (LAN) (IC9), and basal ganglia network (BG) (IC4) (Figure 1). Detailed information of the selected 14 ICs is provided in Supplementary Table 1. To remove the influence of noise, additional postprocessing was performed on the time courses of the selected 14 ICs. Postprocessing steps included: (1) detrending linear, quadratic, and cubic trends; (2) despiking, which was implemented in 3dDespike; (3) regression of the 6 head motion parameters; and (4) low-pass filtering with a high frequency cut-off of 0.15 Hz. The remaining time courses of these 14 ICs were used for further dynamic FC analyses.

To create the static FC matrix of each subject, we calculated pairwise Pearson’s correlations of the 14 RSNs using the postprocessed time courses over the entire rs-fMRI scan, and the temporal correlation coefficients were then converted to *z*-values *via* Fisher’s r-to-z transformation.

### Computation for Dynamic Functional Connectivity

Dynamic FC was calculated using the Temporal Dynamic Functional Network Connectivity Toolbox (version 1.0a) in GIFT software. A sliding window approach was used to detect time-varying changes of FC within the 14 RSNs during the whole rs-fMRI scan. A window length of 22 TRs (i.e., 44 s) was used to segment the entire time courses of rs-fMRI data into a number of rectangle windows, which was convolved with a Gaussian (σ = 3 TRs) function, resulting in a series of tapered windows. The window was slid step-wise by 1 TR along the entire rs-fMRI scan. The window length of 22 TRs was selected based on a previous study (Preti et al., 2017), which suggested that window sizes of 30–60 s were able to successfully capture resting-state dynamic FC fluctuations. In each sling window, a 14 × 14 pairwise covariance matrix was calculated. Furthermore, to promote sparsity in estimation, the L1 norm penalty was implemented in the graphical LASSO framework (repeated 50 times) (Friedman et al., 2008). A Fisher’s r-to-z transformation was used to convert the values in the resulting 14 × 14 pairwise FC matrices into z-scores. To control for the effect of possible covariates, the converted z-scores were residualized with age, gender, education and the mean FD using multiple linear regression.

### Dynamic Functional Connectivity State Analysis

#### Clustering Analysis

To estimate reoccurring FC patterns (states), k-means clustering analysis was used on all windowed 14 × 14 FC matrices for all subjects. The similarity between each FC matrix and the cluster centroids was estimated using the Manhattan distance (L1 distance) function. An optimal number of clusters was determined to be four (*k* = 4) using the elbow criterion (Allen et al., 2014; Supplementary Figure 2). The k-means clustering algorithm was repeated 100 times to obtain unbiased initial cluster centroids. Subsequently, with the estimated four cluster centroids as initial points, all windowed FC matrices of all subjects were clustered into four reoccurring FC states.

#### Functional Connectivity Strength

The subject-specific centroid of each state was computed by calculating the median value of each FC matrix for that state. For the purposes of group comparisons in FC strength at each state, we also calculated the group-specific centroids of the four states by averaging subject-specific centroids of HD patients, non-dialysis patients and HCs, respectively.

#### Temporal Properties

To quantify the temporal properties of dynamic FC states, we calculated three state transition metrics (Kim et al., 2017): (1) fractional window; (2) mean dwell time; and (3) number of transitions. The detailed meaning of these three temporal metrics has been described in previous studies (Allen et al., 2014; Kim et al., 2017).

### Dynamic Graph Properties: Variance in Global Network Metrics

A graph theoretical analysis was used to evaluate the variance in topological organization of the FC, wherein we defined those selected 14 ICs as nodes and the Pearson’s correlation coefficients between each pair of ICs as edges. Construction and calculation of the FC network on FC matrices across subjects were performed using the GRaph thEoreTical Network Analysis Toolbox (GRETNA, version 2.0)^4^ (Wang et al., 2015). All 148 windowed FC matrices of each subject were converted into binarized matrices under a series of sparsity thresholds. The threshold range was evaluated based on the criteria suggested by a previous study (Watts and Strogatz, 1998), and was determined to be 0.2-0.3 with a step size of 0.01. Only positive correlations were considered.

For brain functional networks at each sparsity threshold, we calculated the global network measures, which included: (1) network efficiency [global efficiency (*E*_*glob*_) and local efficiency (*E*_*loc*_)]; and (2) small-world metrics [clustering coefficient (*C*_*p*_), characteristic path length (*L*_*p*_), normalized clustering coefficient (γ), normalized characteristic path length (λ), and small-worldness (σ)]. Uses and interpretations of these network measures have been described in detail previously (Rubinov and Sporns, 2010). We then calculated the area under the curve (AUC) for each network metric within the whole range of sparsity thresholds (0.2–0.3). This strategy has been widely used in previous graph theory-based network studies (Wu et al., 2020a,b; Jin et al., 2021; Yue et al., 2021). Finally, we calculated the variance on the AUC changes of each parameter over time to examine dynamic graph properties of FC network as suggested by a previous study (Yu et al., 2015).

### Validation Analysis

Considering the potential effect of window length on dynamic FC properties, we carried out additional validation analyses to test the consistency and reliability of our results using a window length of 20 TR and a window length of 30 TR, respectively. We calculated Pearson’s correlation coefficients between the cluster centroids under the two different window lengths (window length = 22 TR in the main analysis and window length = 20 TR in the validation analysis; and window length = 22 TR in the main analysis and window length = 30 TR in the validation analysis). If a cluster centroid in the additional analysis showed the highest correlation coefficient with a cluster centroid in the main analysis, they were defined as the same state (Wu et al., 2019).

### Statistical Analysis

#### Normality Test

For quantitative data, Kolmogorov-Smirnov test was used to determine the normality of the data distribution. Data conforming to a normal distribution were expressed as mean ± standard deviation, while data not conforming to a normal distribution were expressed as median and interquartile ranges.

#### Group Differences in Demographic and Clinical Data

Group differences in the demographic and clinical data were analyzed using chi-squared test, one-way analysis of variance (ANOVA), and independent two-sample *t*-test. Bonferroni-corrected *post hoc* comparisons were performed if the ANOVA test showed significant differences. Statistical analysis was performed using SPSS software (version 21.0; IBM Corp., Armonk, NY). Statistical significance was defined as *p* < 0.05.

#### Group Differences in Dynamic Functional Connectivity

Group differences in temporal properties of dynamic FC states and in variances of global network metrics were estimated using one-way ANOVA non-parametric Kruskal-Wallis tests, and one-way analysis of covariance (ANCOVA) was used to determine group differences in dynamic inter-network FC strength [false discovery rate (FDR)-corrected *p* < 0.05]. Age, sex, education, disease duration and the mean FD were set as nuisance covariates. The FDR was also used to correct for multiple comparisons in the *post hoc* analyses (FDR-corrected *p* < 0.05).

#### Relationship to Clinical Variables

Partial correlation analyses (two-tailed) were used to estimate the relationships between dynamic FC parameters showing significant group differences and clinical variables including neuropsychological test results and levels of blood biochemical indicators in patients with ESRD (*p* < 0.05, uncorrected), with age, sex, education, disease duration and the mean FD as covariates.

## Results

### Demographic and Clinical Characteristics

Demographic and clinical characteristics for patients with ESRD and HCs are show in Table 1. There were no significant differences in age (*p* = 0.671), sex (*p* = 0.980), education (*p* = 0.771), or the mean FD (*p* = 0.925) among the three groups. The serum creatinine level of HD patients was significantly higher than that of non-dialysis patients (*p* = 0.002). Regarding cognitive performances, the MMSE, MoCA and SDMT scores of both HD and non-dialysis patients were significantly lower than those of HCs (all *p* < 0.05, Bonferroni-corrected). Both patient groups spent longer time to complete TMT-A and TMT-B than the HC group (all *p* < 0.05, Bonferroni-corrected). Furthermore, HD patients had lower MMSE score (*p* = 0.002) and longer completion time of TMT-B (*p* = 0.021) than non-dialysis patients. No significant differences in MoCA score, SDMT score and completion time of TMT-A were found between the two patient groups.

**TABLE 1:** Demographic and clinical characteristics of the participants.

Characteristics	HD (*n* = 50)	Non-D (*n* = 50)	HC (*n* = 64)	*p*-value	*Post hoc* analyses
**Demographic data**					
Sex (male/female)	27/23	26/24	34/30	0.980^a^	n/a
Age (years)	36.10 ± 9.65	35.60 ± 9.06	34.56 ± 9.50	0.671^c^	–
Education (years)	11.54 ± 2.95	11.86 ± 3.35	11.98 ± 3.53	0.771^c^	–
BMI (kg/m^2^)	21.91 ± 3.55	22.31 ± 3.34	22.81 ± 2.80	0.328^c^	–
Disease duration (months)	30.54 ± 16.97	26.64 ± 14.67	n/a	0.222^b^	n/a
Dialysis duration (months)	18.14 ± 9.05	n/a	n/a		n/a
**Laboratory examinations**					
HDL-C (mmol/L)	1.13 ± 0.34	1.09 ± 0.38	n/a	0.638^b^	n/a
LDL-C (mmol/L)	2.36 ± 0.61	2.46 ± 0.90	n/a	0.520^b^	n/a
Total cholesterol (mmol/L)	3.95 ± 1.00	4.23 ± 1.33	n/a	0.237^b^	n/a
Triglycerides (mmol/L)	1.57 ± 0.70	1.59 ± 0.68	n/a	0.836^b^	n/a
Hemoglobin (g/L)	90.54 ± 23.86	95.00 ± 23.02	n/a	0.344^b^	n/a
Hematocrit (%)	27.65 ± 7.63	29.11 ± 7.32	n/a	0.332^b^	n/a
Serum calcium (mmol/L)	2.10 ± 0.33	2.09 ± 0.25	n/a	0.925^b^	n/a
Serum creatinine (μmol/L)	797.47 ± 199.69	675.42 ± 177.16	n/a	0.002^b^	n/a
Urea (mmol/L)	19.68 ± 7.79	21.59 ± 8.26	n/a	0.239^b^	n/a
Uric acid (μmol/L)	437.51 ± 116.71	446.83 ± 113.81	n/a	0.687^b^	n/a
**Neuropsychological tests**					
MMSE (score)	25.86 ± 2.51	27.20 ± 2.20	28.44 ± 1.07	<0.001 ^c^	HD < Non-D < HC
MoCA (score)	24.80 ± 2.32	25.48 ± 2.27	27.59 ± 1.02	<0.001 ^c^	HD < HC; Non-D < HC
TMT-A (s)	81.64 ± 17.68	75.18 ± 14.29	53.20 ± 14.29	<0.001 ^c^	HD < HC; Non-D < HC
TMT-B (s)	125.04 ± 22.75	115.16 ± 14.64	83.39 ± 16.23	<0.001 ^c^	HD < Non-D < HC
SDMT (score)	42.14 ± 9.50	43.28 ± 8.95	49.80 ± 9.82	<0.001 ^c^	HD < HC; Non-D < HC

*All quantitative data are expressed as mean ± standard deviation; numbers for sex data.*

*^a^The p value was calculated by using chi-square test.*

*^b^The p value was calculated by using independent two-samples t-test.*

*^c^The p value was calculated by one-way analysis of variance.*

*HC, healthy controls; HD, hemodialysis; Non-D, non-dialysis; BMI, body mass index; HDL-C, high-density lipoprotein cholesterol; LDL-C, low-density lipoprotein cholesterol; MMSE, Mini-Mental State Examination; MoCA, Montreal Cognitive Assessment; TMT-A, Trail Making Test A; TMT-B, Trail Making Test B; SDMT, Symbol Digit Modalities Test.*

### Intrinsic Functional Connectivity Networks

Based on the spatial overlap of all ICs with the predefined network templates, 14 ICs corresponding to different subnetworks were selected using GICA: dDMN (IC21), vDMN (IC6), PRE (IC5), AN (IC20), DAN (IC8), pVN (IC22), hVN (IC16), SMN (IC7), aSN (IC1), pSN (IC3), RECN (IC18), LECN (IC15), LAN (IC9), and BG (IC4). The spatial maps of the selected 14 ICs are shown in Figure 1. Group averaged static FC matrix and the top 5% strongest connections over the whole rs-fMRI scan are shown in Supplementary Figure 3.

### Dynamic Functional Connectivity State Analysis

#### Temporal Properties

**FIGURE 2:**
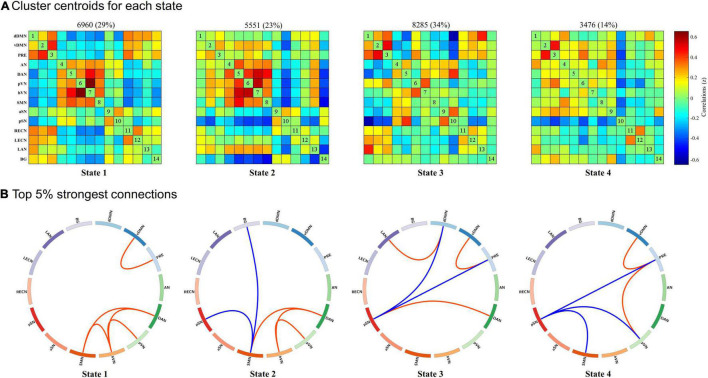
Results of the k-means clustering analysis per state. **(A)** Cluster centroids for each state and the corresponding total number of occurrences and percentage of total occurrences (listed above each cluster median). **(B)** The strongest 5% connections of the functional connectivity (FC) matrix in each state. Each square color represents one of the 14 networks. Red lines represent positive FC, and blue lines represent negative FC. dDMN, dorsal default mode network; vDMN, ventral default mode network; PRE, precuneus network; AN, auditory network; DAN, dorsal attention network; pVN, primary visual network; hVN, higher visual network; SMN, sensorimotor network; aSN, anterior salience network; pSN, posterior salience network; RECN, right executive control network; LECN, left executive control network; LAN, language network; BG, basal ganglia network.

As shown in Figure 2, we identified four highly structured FC states that recurred throughout individual scans and across subjects using k-means clustering algorithm. For better visualization, we kept the strongest 5% connections of each state to clearly show the divergent pattern among FC states. State 1, which accounted for 29% of all windows, was characterized by a strong positive within-network connectivity in DMN (FC between vDMN and PRE) and VN (FC between pVN and hVN) and a strong positive between-networks connectivity in DAN-SMN, DAN-hVN, and SMN-hVN. State 2, which accounted for 23% of all windows, was characterized by a strong positive within-network connectivity in VN (FC between pVN and hVN), a strong positive between-networks connectivity in DAN-SMN and DAN-hVN, and a strong negative between-networks connectivity in SMN-pSN and SMN-BG. State 3, which accounted for 34% of all windows, was characterized by a strong positive within-network connectivity in DMN (FC between vDMN and PRE), a strong positive between-networks connectivity in DAN-pSN and dDMN-LAN, and a strong negative between-networks connectivity in DMN (dDMN and PRE)-pSN. State 4, which accounted for 14% of all windows, was characterized by a strong positive within-network connectivity in DMN (FC between vDMN and PRE), a strong positive between-networks connectivity in pVN-PRE, and a strong negative between-networks connectivity in pSN-PRE, pSN-SMN and pSN-pVN.

**FIGURE 3:**
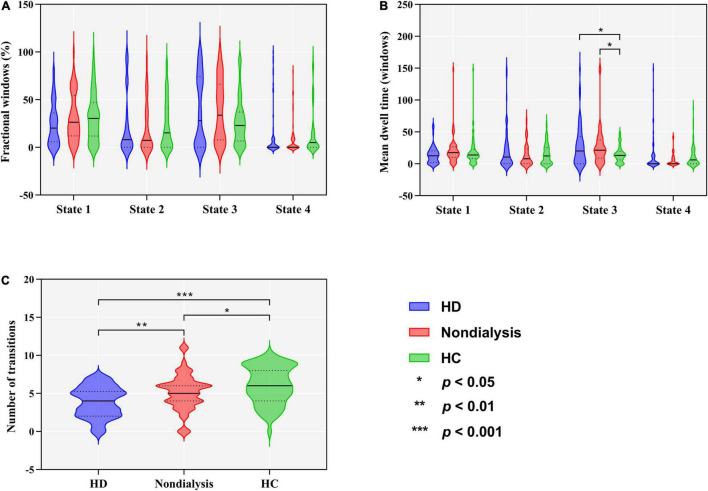
Comparison of temporal properties of dynamic functional connectivity states among the hemodialysis (HD), non-dialysis and healthy control (HC) groups. Violin plots show medians (transverse solid lines) and upper and lower quartiles (transverse dotted lines) of the fractional windows **(A)**, mean dwell time **(B)** and number of transitions **(C)**. *p < 0.05, **p < 0.01, ***p < 0.001.

Group differences in temporal properties are shown in Table 2 and Figure 3. There were significant differences in the mean dwell time in State 3 (*p* = 0.025) and number of transitions (*p* < 0.001) among the three groups. *Post hoc* pairwise comparisons revealed that both HD and non-dialysis patients had a longer mean dwell time in State 3 (*p* = 0.045 and *p* = 0.024, respectively, FDR-corrected) and a higher number of transitions (*p* < 0.001 and *p* = 0.018, respectively, FDR-corrected) than HCs. Compared to non-dialysis patients, HD patients showed a further reduction in the number of transitions across different states (*p* = 0.005, FDR-corrected).

**TABLE 2:** Group differences in temporal properties and variances of global network metrics (window size = 22 TR).

	HD (*n* = 50)		Non-D (*n* = 50)		HC (*n* = 64)		ANOVA	*Post hoc* analyses		
	Median	Interquartile range	Median	Interquartile range	Median	Interquartile range	*p* value	HD vs. HC	Non-D vs. HC	HD vs. Non-D
**Temporal properties**										
**Fractional windows (%)**										
State 1	20.27	(5.91, 36.15)	26.35	(12.16, 54.39)	30.41	(11.99, 47.13)	0.169	–	–	–
State 2	8.11	(0.00, 35.47)	7.43	(0.00, 37.50)	15.20	(0.00, 40.88)	0.790	–	–	–
State 3	28.04	(0.00, 74.32)	33.78	(7.94, 66.22)	22.97	(6.76, 37.16)	0.251	–	–	–
State 4	0.00	(0.00, 10.30)	0.00	(0.00, 9.46)	5.07	(0.00, 19.59)	0.192	–	–	–
**Dwell time (windows)**										
State 1	12.67	(1.88, 20.38)	17.50	(10.00, 26.75)	13.75	(8.25, 22.56)	0.105	–	–	–
State 2	10.50	(0.00, 29.25)	7.75	(0.00, 23.67)	12.25	(0.00, 25.38)	0.751	–	–	–
State 3	20.00	(0.00,42.92)	21.46	(8.75, 37.25)	13.00	(6.17, 19.58)	0.025	0.045	0.024	0.445
State 4	0.00	(0.00, 14.25)	0.00	(0.00, 10.25)	6.00	(0.00, 20.50)	0.178	–	–	–
Number of transitions	4.00	(2.00, 5.25)	5.00	(4.00, 6.00)	6.00	(4.00, 8.00)	<0.001	<0.001	0.018	0.005
**Variance of graph metrics**										
*E*_*glob*_ (× 10^–5^)	3.65	(2.85, 4.67)	3.90	(3.38, 5.00)	4.81	(4.09, 6.21)	<0.001	<0.001	0.001	0.041
*E*_*loc*_ (× 10^–5^)	5.88	(4.61, 7.40)	7.13	(5.41, 8.27)	7.73	(5.99, 9.64)	<0.001	<0.001	0.047	0.047
*C*_*p*_ (× 10^–5^)	6.86	(5.46, 8.52)	7.85	(5.56, 9.46)	7.49	(5.77, 10.04)	0.166	–	–	–
*L*_*p*_ (× 10^–3^)	1.51	(1.05, 2.12)	1.66	(1.25, 2.06)	1.69	(1.14, 2.24)	0.339	–	–	–
γ (× 10^–3^)	3.24	(2.44, 4.37)	3.56	(2.52, 4.84)	3.54	(2.31, 4.96)	0.444	–	–	–
λ (× 10^–4^)	2.94	(2.26, 3.70)	3.17	(2.47, 4.03)	3.35	(2.49, 4.40)	0.261	–	–	–
σ (× 10^–3^)	1.80	(1.48, 2.40)	2.03	(1.64, 2.65)	1.92	(1.25, 3.30)	0.315	–	–	–

*HD, hemodialysis; Non-D, non-dialysis; HC, healthy controls; E_glob_, global efficiency; E_loc_, local efficiency; C_p_, clustering coefficient; L_p_, characteristic path length; γ, normalized clustering coefficient; λ, normalized characteristic path length; σ, small-worldness.*

Additional validation analysis with a window size of 20 TR revealed that the main results remained unchanged. In particular, four dynamic FC states were also identified under this window size across all subjects (Supplementary Figure 4). State 3 under 20 TR window size and State 1 under 22 TR window size (*r* = 0.9998), State 4 under 20 TR window size and State 2 under 22 TR window size (*r* = 1.0000), State 2 under 20 TR window size and State 3 under 22 TR window size (*r* = 0.9998), and State 1 under 20 TR window size and State 4 under 22 TR window size (*r* = 0.9997) showed similar characterization of dynamic FC states (Supplementary Table 2). We found that the main results were reproducible, as significant findings on temporal properties and dynamic graph metrics of the main analysis remained in the validation analysis (Supplementary Table 3).

Additional validation analysis with a window size of 30 TR also revealed that the main results remained unchanged. In particular, four dynamic FC states were also identified under this window size across all subjects (Supplementary Figure 5). State 4 under 30 TR window size and State 1 under 22 TR window size (*r* = 0.9983), State 2 under 30 TR window size and State 2 under 22 TR window size (*r* = 0.9996), State 1 under 30 TR window size and State 3 under 22 TR window size (*r* = 0.9992), and State 3 under 30 TR window size and State 4 under 22 TR window size (*r* = 0.9969) showed similar characterization of dynamic FC states (Supplementary Table 4). We found that the main results were reproducible, as significant findings on temporal properties of the main analysis remained in the validation analysis (Supplementary Table 5).

#### Functional Connectivity Strength

**FIGURE 4:**
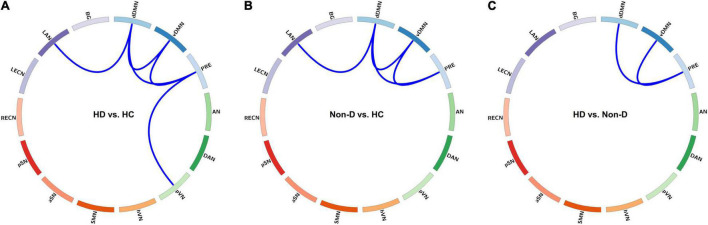
Visualization of group differences in functional connectivity (FC) strength (*z* value) in State 3. Differences in FC strength between the hemodialysis (HD) and healthy control (HC) groups **(A)**, between the non-dialysis (Non-D) and HC groups **(B)**, and between the HD and Non-D groups **(C)** are shown using circular graphs. Blue lines represent decreased FC strength in each pairwise comparison (*p* < 0.05, false discovery rate-corrected). dDMN, dorsal default mode network; vDMN, ventral default mode network; PRE, precuneus network; AN, auditory network; DAN, dorsal attention network; pVN, primary visual network; hVN, higher visual network; SMN, sensorimotor network; aSN, anterior salience network; pSN, posterior salience network; RECN, right executive control network; LECN, left executive control network; LAN, language network; BG, basal ganglia network.

In State 3, both patient groups showed reduced FC within the DMN (involving three default subnetworks, the dDMN, vDMN, and PRE) and between the DMN and LAN compared to the HC group, and a further reduction in FC within the DMN was observed in HD patients compared to non-dialysis patients (all *p* < 0.05, FDR-corrected) (Figure 4). In addition, the HD patients showed lower FC between the PRE and pVN than HCs (*p* = 0.002, FDR-corrected). Comparison of the inter-network FC strength (correlation z value) in State 3 among the three groups is show in Supplementary Figure 6. In the other dynamic FC states, no significant differences in FC strength were observed among the three groups.

### Dynamic Topological Metrics

**FIGURE 5:**
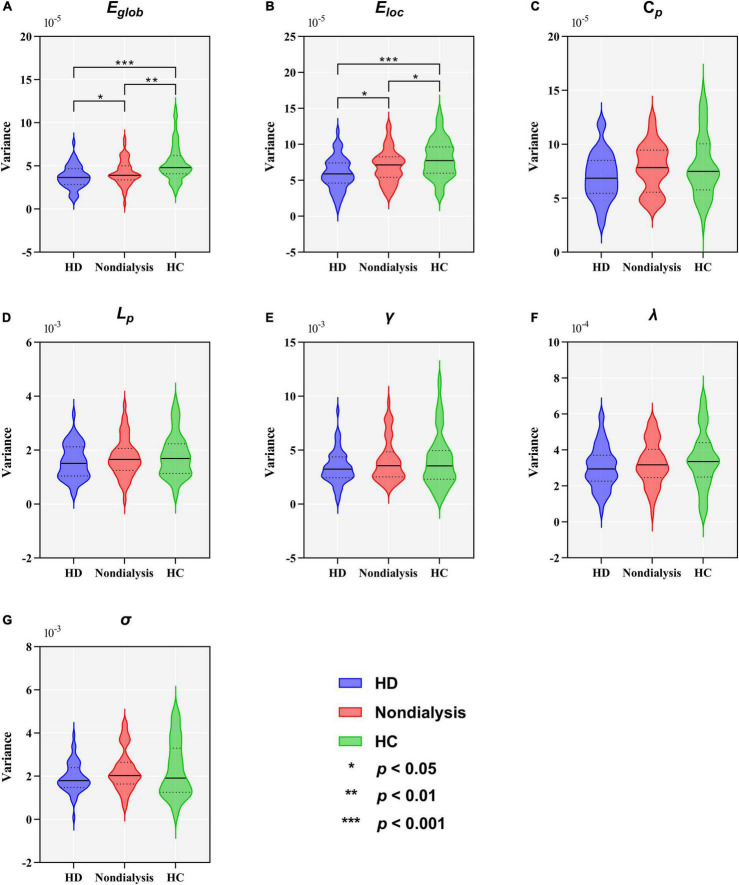
Comparison of variance in global topological metrics among the hemodialysis (HD), non-dialysis and healthy control (HC) groups. Violin plots show medians (transverse solid lines) and upper and lower quartiles (transverse dotted lines) of the variance of the global efficiency (*E*_*glob*_) **(A)**, local efficiency (*E*_*loc*_) **(B)**, clustering coefficient (*C*_*p*_) **(C)**, characteristic path length (*L_*p*_)* **(D)**, normalized clustering coefficient (γ) **(E)**, normalized characteristic path length (λ) **(F)** and small-worldness (σ) **(G)**. *p < 0.05, **p < 0.01, ***p < 0.001.

Table 2 and Figure 5 show group differences in variance of the global network metrics. Both patient groups exhibited lower variance in *E*_*glob*_ and *E*_*loc*_ compared to the HC group (all *p* < 0.05, FDR-corrected). Compared to non-dialysis patients, HD patients showed a further reduction of variance in *E*_*glob*_ (*p* = 0.041, FDR corrected) and *E*_*loc*_ (*p* = 0.047, FDR corrected). These results remained significant in the validation analyses (Supplementary Tables 3, 5). No significant differences in variances of the other graph metrics were found among the three groups.

### Relationship With Clinical Variables

**FIGURE 6:**
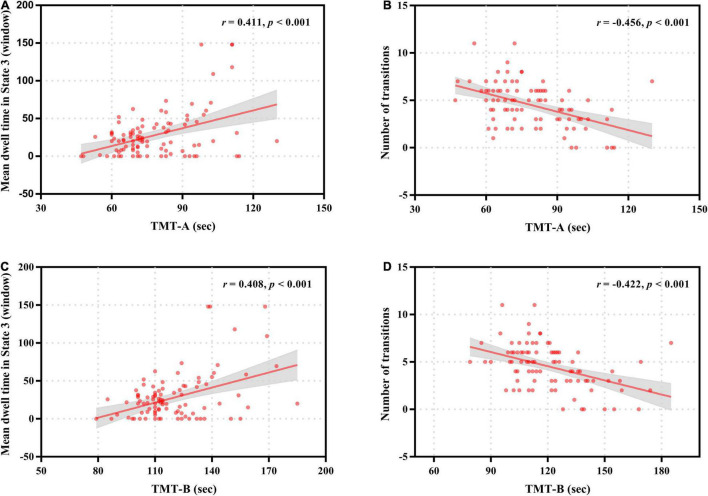
Correlation of neurocognitive test results with temporal properties in dynamic functional connectivity states for patients with end-stage renal disease. The mean dwell time in State 3 was positively correlated with the completion time of Trail Making Test A (TMT-A) **(A)** and Trail Making Test B (TMT-B) **(C)**. The number of transitions was negatively correlated with the completion time of Trail Making Test A (TMT-A) **(B)** and Trail Making Test B (TMT-B) **(D)**.

For patients with ESRD, the mean dwell time of State 3 was positively correlated with the completion time of TMT-A (*r* = 0.411, *p* < 0.001) and TMT-B (*r* = 0.408, *p* < 0.001), and the total number of transitions across states was negatively correlated with the completion time of TMT-A (*r* = –0.456, *p* < 0.001) and TMT-B (*r* = –0.422, *p* < 0.001) (Figure 6). No significant correlations were found between any other dynamic FC properties and clinical variables.

## Discussion

By using rs-fMRI in combination with FC state analysis and graph theory-based network analysis, the present study is the first to investigate the dynamic aspects of whole-brain FC and topological properties in ESRD patients. Our work highlighted that: (1) ESRD patients exhibited impaired functional flexibility of network connections, as characterized by lower number of transitions and lower variance of global network metrics; (2) ESRD patients showed DMN-related FC disruptions in State 3; (3) HD might have an adverse effect on dynamic FC in ESRD patients; (4) significant correlations between altered dynamic FC properties and neurocognitive test results were seen in patients with ESRD. These findings improved our understanding of the pathophysiological mechanisms underlying CI in ESRD patients from the perspective of dynamic FC.

In the present study, patient with ESRD exhibited decreased variance in *E*_*glob*_ and *E*_*loc*_ compared to HCs, suggesting the altered dynamic performance of functional integration and segregation in those patients. In fact, we found an decrease in the total number of transitions across states in patients with ESRD, and the severity of cognitive impairment was associated with the number of transitions in those patients. This finding may explain the lower variance in *E*_*glob*_ and *E*_*loc*_ observed in patients with ESRD, implying abnormal local segregation and global integration of the large-scale brain functional networks in ESRD patients. Dynamic switching between different FC states may increases functional flexibility; thus, this pattern of dynamic networks configuration probably make HCs better adapt to different task demands (Yu et al., 2015). This pattern of dynamic networks configuration may be damaged in patients with ESRD, as they showed a lower total number of transitions across states compared to HCs, and therefore lead to poor cognitive performance. Indeed, the relationship between network flexibility and changing cognitive demands has been demonstrated in previous studies (Spreng and Schacter, 2012; Garrett et al., 2013; Thompson et al., 2013; Madhyastha et al., 2015). Overall, a reduction in the number of transitions and a lower variability in the network *E*_*glob*_ and *E*_*loc*_ indicated impaired functional flexibility of network connections in ESRD patients. Impaired functional flexibility of brain network connectivity may play an important role in the pathophysiology of CI in patients with ESRD.

Similarly, decreased variances of the dynamic graph metrics and total number of state transitions have also been revealed in schizophrenia (Yu et al., 2015) and idiopathic generalized epilepsy with generalized tonic–clonic seizure (Liu et al., 2017). On the contrary, increased total number of transitions and higher variance in graph metrics were demonstrated in some other brain diseases, such as Parkinson’s disease (Kim et al., 2017) and bipolar disorder (Wang et al., 2019), and these alterations were suggested to represent the clinical symptoms of those brain diseases. These similar and opposite findings to our research suggest that altered FC properties may characterize different pathophysiological mechanisms in different diseases.

Interestingly, our study found that patients with ESRD had aberrant inter-network connections only in State 3, suggesting that the network connectivity disruptions in patients with ESRD might be state-dependent. Notably, the disrupted FC was mainly related to the DMN, especially the FC reductions between the DMN subnetworks (i.e., the dDMN, vDMN, and PRE). Core regions of the DMN mainly include the ventral medial prefrontal cortex, dorsal medial prefrontal cortex, posterior cingulate cortex/precuneus and inferior parietal lobule, which are engaged in multiple cognitive functions including memory, visual and auditory attention, motor activity, and language processing (Raichle et al., 2001; Buckner et al., 2008; Raichle, 2015). Convergent evidences from functional neuroimaging studies demonstrated high spatial overlap of the functional hubs with regions of the DMN, indicating a critical role of the DMN in the overall network structure (van den Heuvel and Sporns, 2013; Liu et al., 2015). Furthermore, a recent study suggests that the DMN is composed of subnetworks that exhibit differential task engagement, and the DMN subnetworks interact in a dynamic equilibrium, which is crucial for the maintenance of normal cognition (Gordon et al., 2020). Thus, our findings of reduced FC between the DMN subnetworks in patients with ESRD might suggest abnormal functional integration of core regions in their DMN, which might underlie the impairments of DMN-related cognitive domains including memory, concentration, executive function, attention and language processing in these patients. In fact, these speculations were supported to some extent by our other findings that patients with ESRD spent longer time in State 3 and the mean dwell time in State 3 was positively correlated with the completion time of TMT-A and TMT-B (the longer the completion time, the worse the cognitive dysfunction), which are widely used to measure concentration, attention, executive function and processing speed (Corrêa et al., 2016; Cook et al., 2017). These findings on FC strength of dynamic states together with correlation analysis results provided crucial information for better understanding the neural bases of CI in patients with ESRD.

Functional abnormalities in the DMN have been well demonstrated in patients with ESRD by previous neuroimaging studies. Using an amplitude of low-frequency fluctuation (ALFF) algorithm, Luo et al. (2016) reported that patients with ESRD exhibited impaired spontaneous brain activity in the DMN regions, including the left superior parietal lobe, left inferior parietal lobe and left precuneus. Based on ICA or seed-based methods, some other studies also demonstrated disrupted FC within the DMN in patients with ESRD (Ni et al., 2014; Ma et al., 2016; Lu et al., 2019). These functional impairments of the DMN regions may influence the normal interactions between DMN subnetworks. Indeed, a recent study did demonstrate that patients with ESRD exhibit significantly lower FC between the two DMN hubs (i.e., the posterior cingulate cortex (PCC) and the anterior medial prefrontal cortex) and between the two DMN subnetworks (i.e., the dorsal medial prefrontal cortex subnetwork and the medial temporal lobe subnetwork) compared to HCs (Ma et al., 2020). Evidences from these studies suggest functional deficits of the DMN regions and abnormal functional interactions of the DMN subnetworks, and thus support our findings.

We also found reduced FC between the dDMN and LAN in patients with ESRD compared to HCs. The DMN can integrate information from primary function and cognition networks (Liao et al., 2014). Functional abnormalities of the DMN mentioned above may also affect its information communication with other brain networks, such as the LAN, thus resulting in impaired functional integrations between the DMN subnetworks and LAN. The LAN is mainly associated with various language functions (Friederici and Gierhan, 2013). The DMN is also engaged in language comprehension (Tesink et al., 2009), and core hubs of the dDMN such as medial prefrontal cortex and angular gyrus can predicts language processing in healthy adults (Tomasi and Volkow, 2020). Thus, our findings may suggest impaired dDMN-LAN interactions involving language processing and may explain the deficits in language function observed in these patients (Arnold et al., 2016).

The present study found a further reduction in the number of transitions across states, the variance of *E*_*glob*_ and *E*_*loc*_, and the FC strength between the DMN subnetworks of State 3 in HD patients compared to non-dialysis patients, suggesting that HD may have an adverse effect on dynamic FC. Notably, comparison of the neuropsychological test results also revealed a worse cognitive performance in HD patients than non-dialysis patients. Dialysis may exert deleterious effects on the brain, over and above that of chronic kidney disease, and dialysis initiation is associated with loss of executive function (Kurella Tamura et al., 2017). In addition, dialysis patients have been shown to have a high cerebrovascular burden, regardless of dialysis modality (Kim et al., 2011). Potential mechanisms by which dialysis affects the brain include intradialytic hypotension, reduced cerebra blood flow, inflammation and oxidative stress (Iyasere and Brown, 2017).

Consistent with our study, previous neuroimaging studies also demonstrated that HD patients exhibited further deficits in brain function than non-dialysis patients. For example, Chen et al. (2015) reported that HD patients showed lower regional homogeneity mainly in the DMN regions than non-dialysis patients, and these abnormalities were associated with CI in HD patients. Another study investigated the intrinsic brain activity in patients with ESRD who underwent peritoneal dialysis (PD), and found that PD patients showed lower ALFF values in the DMN regions compared to non-dialysis patients. Our recent study also revealed that HD patients had more severe disrupted whole-brain functional network than non-dialysis patients, and dialysis duration was associated with *E*_*glob*_ in HD patients, suggesting that HD may be an independent factor for impaired ability of global network integration (Wu et al., 2020a). Our findings combined with those from previous studies may have some clinical implications. As our understanding of the effect of HD on brain function improves, novel dialysis strategies may be developed to reduce the adverse effects of HD on the brain. In fact, a previous randomized control trial found that HD caused obvious brain injury, and after improvement of hemodynamic tolerability by using cooled dialysate, this adverse impact could be effectively abrogated (Eldehni et al., 2015).

Several limitations should be acknowledged in our study. First, this was a cross-sectional study with a relatively small sample size, which may affect the statistical power. Future studies with a longitudinal design and larger sample sizes are needed to further evaluate the dynamic FC changes in patients with ESRD before and after HD treatment. Second, in dynamic FC analysis, parameter setting is still controversial, and the gold standard has not been established. However, we conducted additional validation analyses to test the reproducibility of the results, and found that the main results remained unchanged. Finally, in our study, each functional dataset only contained 180 timepoints. This might affect the evaluation of dynamic FC. Future studies with longer timepoints of rs-fMRI data should be conducted to verify our findings.

## Conclusion

The present study demonstrated abnormal dynamic FC in patients with ESRD, characterized by increased time in State 3, lower number of transitions across states, lower variance in network efficiency and disrupted FC related to the DMN in State 3, and some altered time-varying metrics were associated with cognitive performance. Moreover, these abnormalities of dynamic FC were more severe in HD patients than in non-dialysis patients. These findings suggest impaired functional flexibility of network connections and state-specific FC disruptions in patients with ESRD, which may provide new insights into the pathophysiological mechanisms underlying their cognitive deficits from the perspective of dynamic FC.

## Data Availability Statement

The original contributions presented in the study are included in the article/Supplementary Material, further inquiries can be directed to the corresponding author/s.

## Ethics Statement

The studies involving human participants were reviewed and approved by the Institutional Review Board of the First Affiliated Hospital of Xinxiang Medical University. The patients/participants provided their written informed consent to participate in this study.

## Author Contributions

BW conceptualized the project. BW and XL designed the study and drafted the manuscript. XL, RY, ZY, MZ, and JR contributed to data acquisition. BW and XL contributed to literature search, data analysis, and data interpretation. BW critically revised the manuscript. All authors approved the final version of the manuscript.

## Conflict of Interest

The authors declare that the research was conducted in the absence of any commercial or financial relationships that could be construed as a potential conflict of interest.

## Publisher’s Note

All claims expressed in this article are solely those of the authors and do not necessarily represent those of their affiliated organizations, or those of the publisher, the editors and the reviewers. Any product that may be evaluated in this article, or claim that may be made by its manufacturer, is not guaranteed or endorsed by the publisher.

## Footnotes

1
http://rfmri.org/dpabi

2
http://mialab.mrn.org/software/gift

3
http://findlab.stanford.edu/functional_ROIs.html

4
http://www.nitrc.org/projects/gretna
