# Supplementary material for: Abnormal Dynamic Functional Connectivity in Patients With End-Stage Renal Disease
Source: Front Neurosci. 2022 May 20;16:852822. doi: 10.3389/fnins.2022.852822 (PMC9163405; doi:10.3389/fnins.2022.852822)
Supplement: Supplementary file 1 [file Data_Sheet_1.docx]

***Supplementary Materials***

**Table S1.** Information of the 14 identified independent components and their spatial overlap with the predefined template.

| Intrinsic connectivity network | IC number | *I*_q_ | Postprocessing results | |  | Peak coordinate  (MNI) | | | *R*^2^ |
| --- | --- | --- | --- | --- | --- | --- | --- | --- | --- |
|  |  |  | Dynamic range | Power_LF_/Power_HF_ |  | *x* | *y* | *z* |  |
| Dorsal default mode network (dDMN) | 21 | 0.982 | 0.051 | 4.309 |  | 0 | 56 | 20 | 0.342 |
| Ventral default mode network (vDMN) | 6 | 0.985 | 0.053 | 4.728 |  | -6 | -56 | 6 | 0.215 |
| Precuneus network (PRE) | 5 | 0.980 | 0.050 | 4.598 |  | 0 | -76 | 36 | 0.361 |
| Auditory network (AN) | 20 | 0.986 | 0.047 | 2.551 |  | 62 | -18 | 8 | 0.182 |
| Dorsal attention network (DAN) | 8 | 0.980 | 0.055 | 5.576 |  | 42 | -42 | 66 | 0.110 |
| Primary visual network (pVN) | 22 | 0.977 | 0.050 | 4.888 |  | 0 | -84 | 20 | 0.215 |
| Higher visual network (hVN) | 16 | 0.974 | 0.049 | 4.328 |  | 36 | -86 | 6 | 0.233 |
| Sensorimotor network (SMN) | 7 | 0.981 | 0.047 | 3.790 |  | 60 | -4 | 26 | 0.093 |
| Anterior SN (aSN) | 1 | 0.975 | 0.051 | 4.353 |  | 32 | 54 | 24 | 0.232 |
| Posterior SN (pSN) | 3 | 0.973 | 0.054 | 5.177 |  | 62 | -30 | 36 | 0.254 |
| Right executive control network (RECN) | 18 | 0.972 | 0.051 | 4.501 |  | 48 | -62 | 48 | 0.306 |
| Left executive control network (LECN) | 15 | 0.968 | 0.049 | 4.502 |  | -42 | -66 | 50 | 0.394 |
| Language network (LAN) | 9 | 0.975 | 0.050 | 4.597 |  | 60 | -52 | 16 | 0.240 |
| Basal ganglia network (BG) | 4 | 0.967 | 0.032 | 2.229 |  | -4 | 4 | 2 | 0.086 |

*Note: R*^2^ represents the coefficient of determination of each IC for a specific network of the Stanford functional ROI template (findlab.stanford.edu/functional_ROIs.html). Dynamic range represents difference between the peak power and minimum power at frequencies to the right of the peak. Power_LF_/Power_HF_ represents low frequency (LF) to high frequency (HF) power ratio. IC, independent component; MNI, Montreal Neurological Institute; I_q_, quality index.

**Table S2.** Correlation coefficients between pairs of cluster centroids under different window sizes.

| Window size = 22 TR | Window size = 20 TR | | | |
| --- | --- | --- | --- | --- |
|  | State 1 | State 2 | State 3 | State 4 |
| State 1 | *r* = 0.3207 | *r* = 0.6796 | *r* = 0.9998^*^ | *r* = 0.5075 |
| State 2 | *r* = 0.4317 | *r* = 0.3497 | *r* = 0.4989 | *r* = 1.0000^*^ |
| State 3 | *r* = 0.3639 | *r* = 0.9998^*^ | *r* = 0.6879 | *r* = 0.3500 |
| State 4 | *r* = 0.9997^*^ | *r* = 0.3591 | *r* = 0.3183 | *r* = 0.4229 |

^*^ Represents the highest similarity of state centroids under the two different window sizes.

|  | HD (*n* = 50) | |  | Non-D (*n* = 50) | |  | HC (*n* = 64) | | ANOVA |  | Post-hoc analyses | | |
| --- | --- | --- | --- | --- | --- | --- | --- | --- | --- | --- | --- | --- | --- |
|  | Median | Interquartile  range |  | Median | Interquartile range |  | Median | Interquartile range | *p* value |  | HD vs. HC | Non-D vs. HC | HD vs. Non-D |
| Temporal properties |  |  |  |  |  |  |  |  |  |  |  |  |  |
| Fractional windows (%) |  |  |  |  |  |  |  |  |  |  |  |  |  |
| State 1 | 0.00 | (0.00, 9.33) |  | 1.67 | (0.00, 17.50) |  | 7.00 | (0.00, 14.50) | 0.191 |  | ‒ | ‒ | ‒ |
| State 2 | 33.00 | (2.83, 74.50) |  | 25.67 | (9.17, 63.00) |  | 23.67 | (4.83, 41.00) | 0.239 |  | ‒ | ‒ | ‒ |
| State 3 | 18.67 | (2.50, 46.67) |  | 25.33 | (9.00, 37.33) |  | 31.00 | (11.67, 52.00) | 0.121 |  | ‒ | ‒ | ‒ |
| State 4 | 10.67 | (0.00, 30.17) |  | 8.67 | (0.00, 40.67) |  | 13.33 | (0.00, 42.17) | 0.556 |  | ‒ | ‒ | ‒ |
| Dwell time (windows) |  |  |  |  |  |  |  |  |  |  |  |  |  |
| State 1 | 0.00 | (0.00, 13.13) |  | 2.50 | (0.00, 17.65) |  | 7.50 | (0.00, 12.75) | 0.278 |  | ‒ | ‒ | ‒ |
| State 2 | 20.75 | (4.25, 39.83) |  | 16.04 | (9.88, 32.38) |  | 13.00 | (4.50, 20.50) | 0.026 |  | 0.012 | 0.012 | 0.346 |
| State 3 | 12.13 | (3.38, 24.75) |  | 14.00 | (7.23, 20.33) |  | 13.38 | (8.50, 20.28) | 0.789 |  | ‒ | ‒ | ‒ |
| State 4 | 10.00 | (0.00, 26.25) |  | 5.92 | (0.00, 23.33) |  | 10.75 | (0.00, 25.75) | 0.501 |  | ‒ | ‒ | ‒ |
| Number of transitions | 4.00 | (3.00, 6.00) |  | 5.00 | (4.00, 7.00) |  | 7.00 | (6.00, 9.00) | < 0.001 |  | < 0.001 | 0.014 | 0.016 |
| Variance of graph metrics |  |  |  |  |  |  |  |  |  |  |  |  |  |
| *E_glob_* (×10^-5^) | 3.59 | (2.70, 4.74) |  | 3.93 | (3.19, 5.24) |  | 4.78 | (4.07, 6.39) | < 0.001 |  | < 0.001 | 0.001 | 0.044 |
| *E_loc_* (×10^-5^) | 5.97 | (4.79, 7.28) |  | 6.89 | (5.55, 8.34) |  | 7.92 | (6.17, 10.32) | < 0.001 |  | < 0.001 | 0.043 | 0.035 |
| *C_p_* (×10^-5^) | 7.05 | (5.60, 8.50) |  | 7.33 | (4.77, 8.98) |  | 7.53 | (6.09, 9.79) | 0.244 |  | ‒ | ‒ | ‒ |
| *L_p_* (×10^-3^) | 1.51 | (1.06, 2.12) |  | 1.52 | (1.15, 2.01) |  | 1.76 | (1.11, 2.24) | 0.367 |  | ‒ | ‒ | ‒ |
| *γ* (×10^-3^) | 3.12 | (2.42, 4.48) |  | 3.43 | (2.53, 4.83) |  | 3.77 | (2.31, 4.92) | 0.400 |  | ‒ | ‒ | ‒ |
| *λ* (×10^-4^) | 2.82 | (2.18, 3.64) |  | 3.20 | (2.36, 4.45) |  | 3.34 | (2.25, 4.46) | 0.269 |  | ‒ | ‒ | ‒ |
| *σ* (×10^-3^) | 2.00 | (1.55, 2.65) |  | 2.09 | (1.64, 2.84) |  | 2.28 | (1.26, 3.49) | 0.496 |  | ‒ | ‒ | ‒ |

**Table S3.** Group differences in temporal properties and variances of global network metrics (window size = 20 TR)

*Note:* Our validation analyses (window size = 20 TR) found that both patient groups had significantly higher mean dwell time in State 2 than the HC group, which was equivalent to the statistical results of State 3 in the main analyses (window size = 22 TR). Validation analyses also found similar statistical results in terms of number of transitions and variance of graph metrics, suggesting a consistency and reliability of our main findings. HD, hemodialysis; Non-D, non-dialysis; HC, healthy controls; E_glob_, global efficiency; E_loc_, local efficiency; C_p_, clustering coefficient; L_p_, characteristic path length; γ, normalized clustering coefficient; λ, normalized characteristic path length; σ, small-worldness

**Table S4.** Correlation coefficients between pairs of cluster centroids under different window sizes.

| Window size = 22 TR | Window size = 30 TR | | | |
| --- | --- | --- | --- | --- |
|  | State 1 | State 2 | State 3 | State 4 |
| State 1 | *r* = 0.6985 | *r* = 0.5222 | *r* = 0.3257 | *r* = 0.9983^*^ |
| State 2 | *r* = 0.3593 | *r* = 0.9996^*^ | *r* = 0.4231 | *r* = 0.5349 |
| State 3 | *r* = 0.9992^*^ | *r* = 0.3612 | *r* = 0.3761 | *r* = 0.7094 |
| State 4 | *r* = 0.3725 | *r* = 0.4367 | *r* = 0.9969^*^ | *r* = 0.3339 |

^*^ Represents the highest similarity of state centroids under the two different window sizes.

**Table S5.** Group differences in temporal properties and variances of global network metrics (window size = 30 TR)

|  | HD (*n* = 50) | |  | Non-D (*n* = 50) | |  | HC (*n* = 64) | | ANOVA |  | Post-hoc analyses | | |
| --- | --- | --- | --- | --- | --- | --- | --- | --- | --- | --- | --- | --- | --- |
|  | Median | Interquartile  range |  | Median | Interquartile range |  | Median | Interquartile range | *p* value |  | HD vs. HC | Non-D vs. HC | HD vs. Non-d |
| Temporal properties |  |  |  |  |  |  |  |  |  |  |  |  |  |
| Fractional windows (%) |  |  |  |  |  |  |  |  |  |  |  |  |  |
| State 1 | 27.14 | (0.00, 83.04) |  | 33.21 | (6.79, 72.14) |  | 14.64 | (0.00, 35.54) | 0.057 |  | ‒ | ‒ | ‒ |
| State 2 | 0.71 | (0.00, 28.75) |  | 5.36 | (0.00, 33.93) |  | 12.14 | (0.00, 56.61) | 0.159 |  | ‒ | ‒ | ‒ |
| State 3 | 0.00 | (0.00, 6.79) |  | 0.00 | (0.00, 6.61) |  | 0.36 | (0.00, 20.89) | 0.227 |  | ‒ | ‒ | ‒ |
| State 4 | 16.07 | (0.00, 43.57) |  | 23.93 | (6.43, 45.89) |  | 27.14 | (7.86, 51.79) | 0.291 |  | ‒ | ‒ | ‒ |
| Dwell time (windows) |  |  |  |  |  |  |  |  |  |  |  |  |  |
| State 1 | 25.75 | (0.00, 52.63) |  | 23.5 | (8.00, 41.75) |  | 9.75 | (0.00, 21.75) | 0.014 |  | 0.008 | 0.008 | 0.331 |
| State 2 | 1.00 | (0.00, 25.50) |  | 6.00 | (0.00, 28.63) |  | 11.00 | (0.00, 33.83) | 0.382 |  | ‒ | ‒ | ‒ |
| State 3 | 0.00 | (0.00, 9.50) |  | 0.00 | (0.00, 9.25) |  | 0.50 | (0.00, 18.25) | 0.253 |  | ‒ | ‒ | ‒ |
| State 4 | 12.00 | (0.00, 28.63) |  | 20.75 | (8.58, 30.54) |  | 14.67 | (6.63, 22.75) | 0.320 |  | ‒ | ‒ | ‒ |
| Number of transitions | 3.00 | (1.00, 4.00) |  | 3.00 | (2.00, 4.25) |  | 5.00 | (3.00, 6.75) | < 0.001 |  | < 0.001 | 0.006 | 0.018 |
| Variance of graph metrics |  |  |  |  |  |  |  |  |  |  |  |  |  |
| *E_glob_* (×10^-5^) | 3.44 | (1.97, 5.53) |  | 4.60 | (2.23, 6.77) |  | 6.65 | (3.18, 11.95) | < 0.001 |  | < 0.001 | 0.013 | 0.043 |
| *E_loc_* (×10^-5^) | 4.96 | (3.33, 6.93) |  | 6.37 | (4.16, 11.15) |  | 8.91 | (5.35, 13.97) | < 0.001 |  | < 0.001 | 0.045 | 0.041 |
| *C_p_* (×10^-5^) | 7.53 | (5.78, 9.63) |  | 7.83 | (5.22, 10.03) |  | 7.40 | (5.63, 9.34) | 0.950 |  | ‒ | ‒ | ‒ |
| *L_p_* (×10^-3^) | 1.14 | (0.46, 3.83) |  | 1.36 | (0.63, 3.58) |  | 1.48 | (0.75, 3.39) | 0.354 |  | ‒ | ‒ | ‒ |
| *γ* (×10^-3^) | 6.22 | (4.70, 8.55) |  | 5.24 | (3.73, 7.69) |  | 5.48 | (4.17, 7.51) | 0.228 |  | ‒ | ‒ | ‒ |
| *λ* (×10^-4^) | 0.98 | (0.56, 2.91) |  | 1.45 | (0.69, 2.44) |  | 1.36 | (0.69, 2.81) | 0.549 |  | ‒ | ‒ | ‒ |
| *σ* (×10^-3^) | 5.45 | (3.96, 7.76) |  | 5.00 | (3.40, 6.82) |  | 4.70 | (3.49, 6.96) | 0.324 |  | ‒ | ‒ | ‒ |

*Note:* Our validation analyses (window size = 30 TR) found that both patient groups had significantly higher mean dwell time in State 1 than the HC group, which was equivalent to the statistical results of State 3 in the main analyses (window size = 22 TR). Validation analyses also found similar statistical results in terms of number of transitions and variance of graph metrics, suggesting a consistency and reliability of our main findings. HD, hemodialysis; Non-D, non-dialysis; HC, healthy controls; E_glob_, global efficiency; E_loc_, local efficiency; C_p_, clustering coefficient; L_p_, characteristic path length; γ, normalized clustering coefficient; λ, normalized characteristic path length; σ, small-worldness


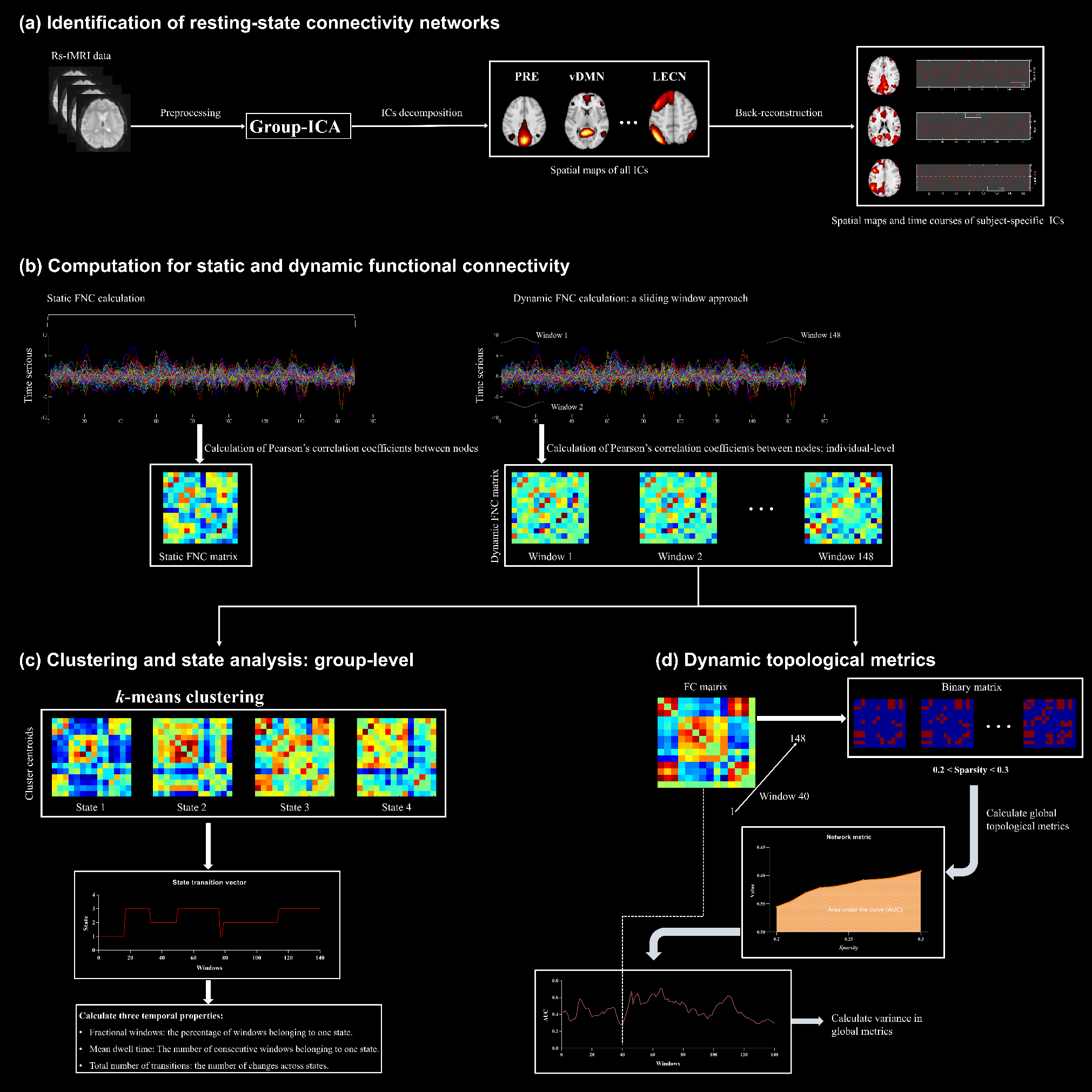
**Figure S1.** A flowchart of the dynamic functional connectivity (FC) analysis steps used in this study. A brief description was as follows: (a) 23 independent components (ICs) were obtained using group independent component analysis, and 14 of 23 ICs were identified to correspond to different resting-state networks (RSNs) based on a predefined template; static FC matrix was constructed by calculating the Pearson’s correlation coefficients of time courses between each pair of RSNs over the entire resting state scan, and dynamic FC was calculated using a sliding-window approach; (c) three dynamic FC states were identified using k-means clustering algorithm, and the temporal properties of dynamic FC states were calculated; (d) for the dynamic graph theory analysis, each FC matrix in each window was binarized with a range of sparsity thresholds, and then the variance on the changes of the area under curve of global network metrics over time was calculated for each subject.


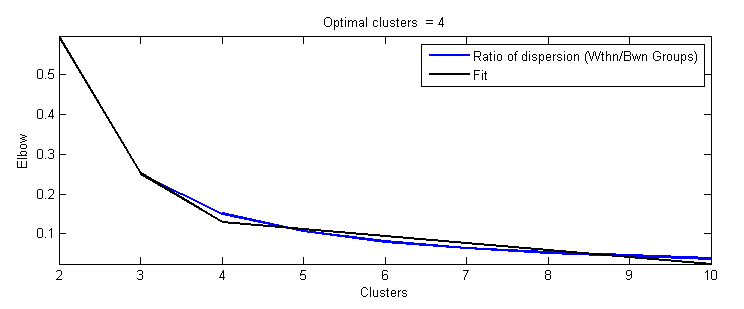
**Figure S2.** Elbow criterion for k-means clustering algorithm. The blue curve shows the observed values of the average within-cluster sum of square for cluster size, k = 2 to 10. The black curve shows the best fit of the elbow-shaped curve to the observed data (blue curve), by minimizing the distance between the observed data and the elbow-shaped curve. Here, the optimum number of clusters is 4 as shown by the elbow-shaped curve.


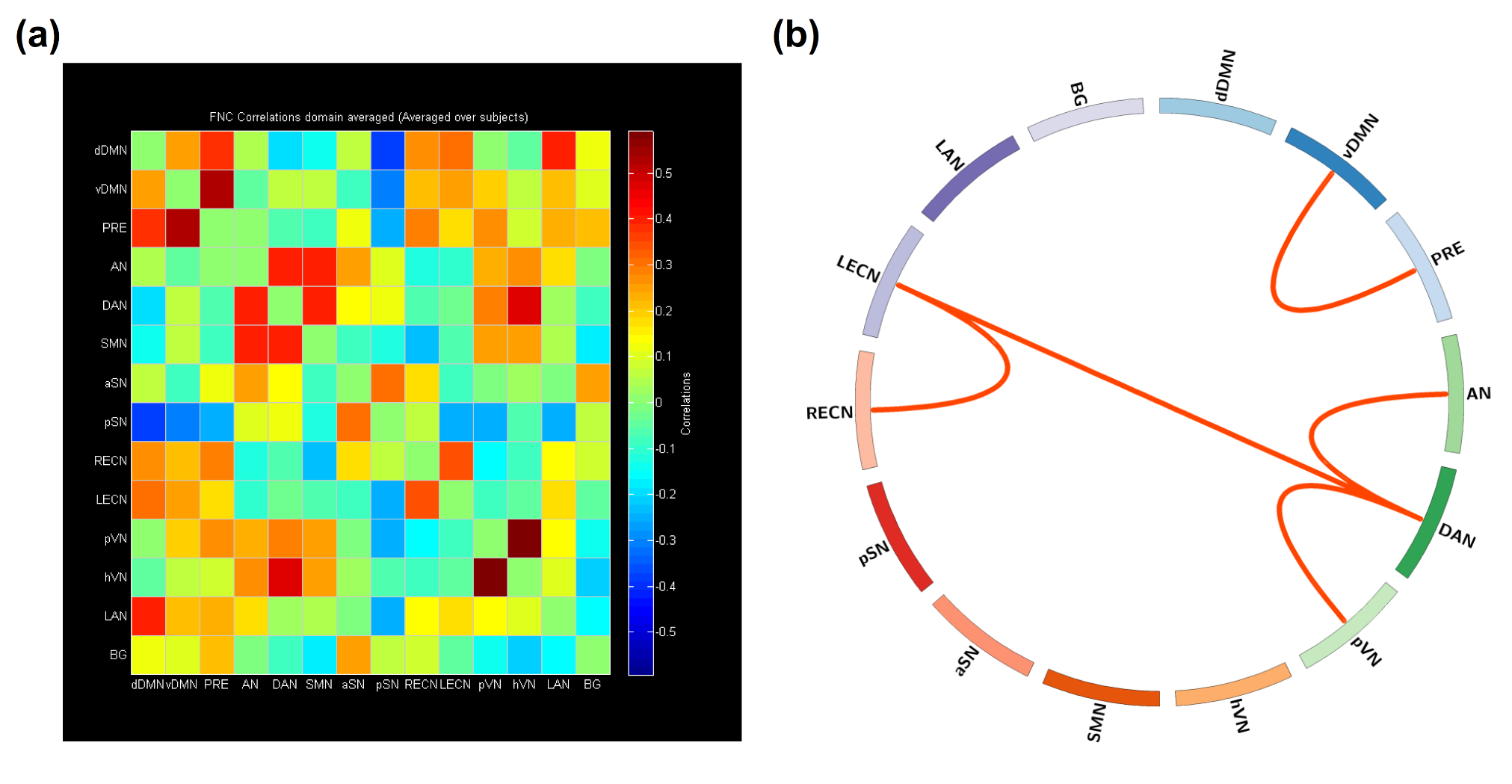
**Figure S3.** Group averaged static functional connectivity matrix (a) and the top 5% strongest connections (b) over the entire resting state scan. dDMN, dorsal default mode network; vDMN, ventral default mode network; PRE, precuneus network; AN, auditory network; DAN, dorsal attention network; pVN, primary visual network; hVN, higher visual network; SMN, sensorimotor network; aSN, anterior salience network; pSN, posterior salience network; RECN, right executive control network; LECN, left executive control network; LAN, language network; BG, basal ganglia network.


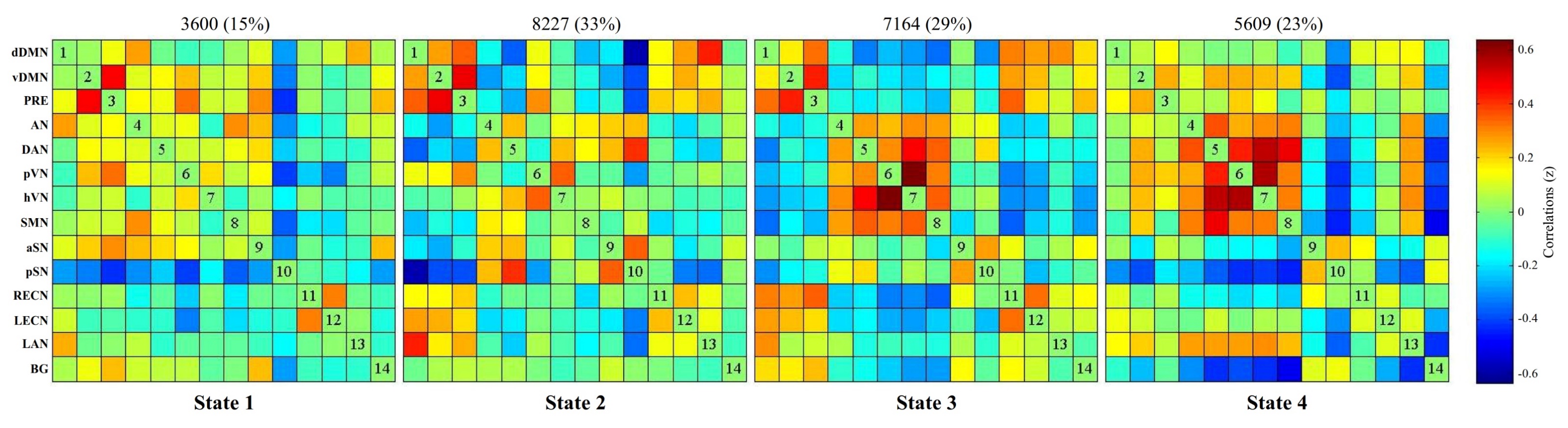
**Figure S4.** Cluster centroids for each state under the window size of 20 TR. The total number of occurrences and percentage of total occurrences are listed above each cluster median. dDMN, dorsal default mode network; vDMN, ventral default mode network; PRE, precuneus network; AN, auditory network; DAN, dorsal attention network; pVN, primary visual network; hVN, higher visual network; SMN, sensorimotor network; aSN, anterior salience network; pSN, posterior salience network; RECN, right executive control network; LECN, left executive control network; LAN, language network; BG, basal ganglia network.


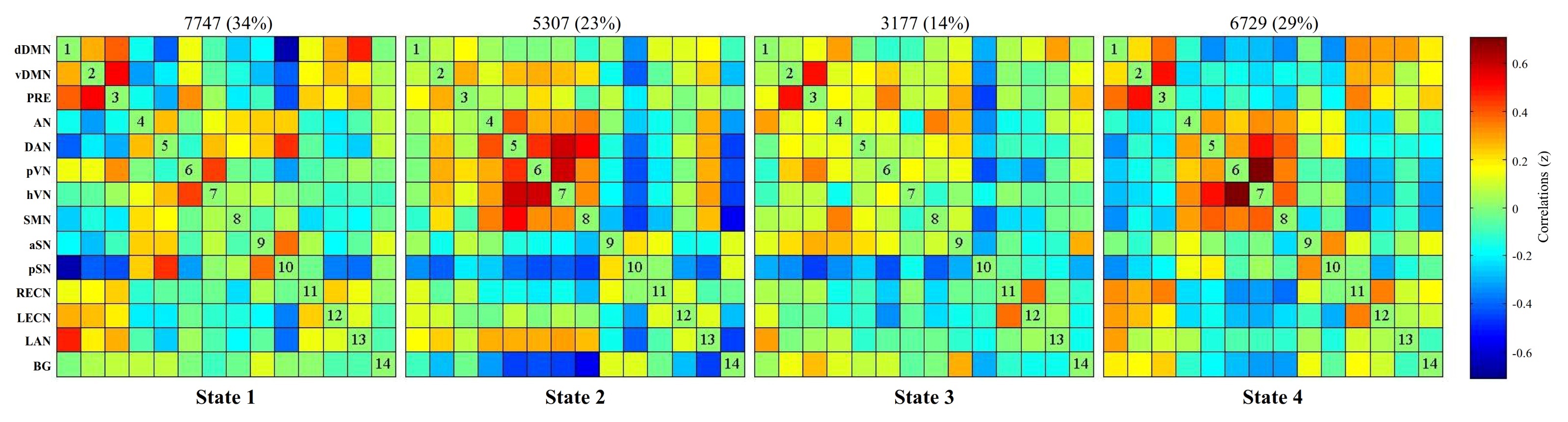
**Figure S5.** Cluster centroids for each state under the window size of 30 TR. The total number of occurrences and percentage of total occurrences are listed above each cluster median. dDMN, dorsal default mode network; vDMN, ventral default mode network; PRE, precuneus network; AN, auditory network; DAN, dorsal attention network; pVN, primary visual network; hVN, higher visual network; SMN, sensorimotor network; aSN, anterior salience network; pSN, posterior salience network; RECN, right executive control network; LECN, left executive control network; LAN, language network; BG, basal ganglia network.


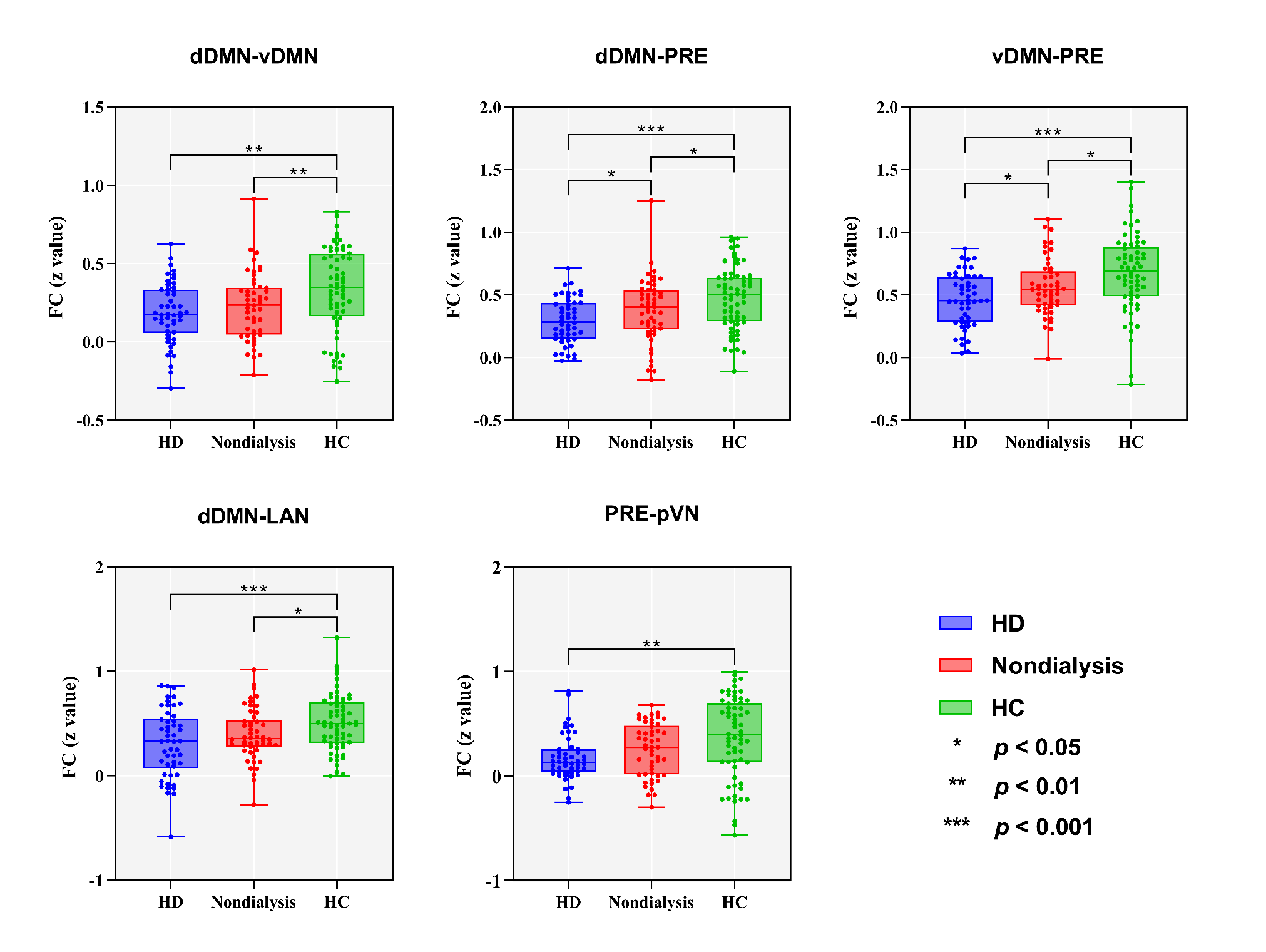
**Figure S6.** Differences in the inter-network functional connectivity (FC) strength in State 3 among the hemodialysis (HD), non-dialysis and healthy control (HC) groups. Box plots show the individual data points, minimum value, lower quartile, median, upper quartile and maximum value. dDMN, dorsal default mode network; vDMN, ventral default mode network; PRE, precuneus network; LAN, language network; pVN, primary visual network. **p* < 0.05, ***p* < 0.01, ****p* < 0.001.
